# Aqueous and pH dependent coacervation method for taste masking of paracetamol via amorphous solid dispersion formation

**DOI:** 10.1038/s41598-021-88312-6

**Published:** 2021-04-26

**Authors:** Basheer Al-kasmi, M. H. D. Bashir Alsirawan, Anant Paradkar, Abdul-Hakim Nattouf, Hind El-Zein

**Affiliations:** 1 Department of Pharmaceutics and Pharmaceutical Technology, Faculty of Pharmacy, Damascus University, Damascus, Syria; 2 Center for Pharmaceutical Engineering Science, University of Bradford, Bradford, UK

**Keywords:** Medical research, Drug development, Materials science, Techniques and instrumentation

## Abstract

Taste masking of paracetamol was achieved by preparing amorphous solid dispersion (ASD) using modified coacervation method. The method is based on dissolving the drug and polymeric carrier in water adjusted to certain pH level. Then, precipitation of ASD granules is performed by gradually changing pH level. Therefore, the chosen drug and polymer should obtain appropriate acidic or basic groups to enable pH-dependent solvation. Moreover, using solubility enhancing additives such as sodium lauryl sulphate (SLS) and low viscosity polyethylene glycol (PEG 400) found to be essential in aiding drug/polymer aqueous solvation which enhanced amorphization, hence taste masking and drug loading. Solid dispersion between Paracetamol and Eudragit E was formed and that proved by FT-IR, DSC, PXRD and SEM. Also, Paracetamol was released after 2 min in 0.1 N hydrochloric acid medium and the taste of masking forms are accepted from all volunteers. Modified coacervation method does not involve organic solvents, high temperatures, or sophisticated instruments commonly used in taste masking methods. Using PEG 400 resulted in significantly higher drug loading and dissolution rate compared to SLS granules. Moreover, using previously reported scoring system for the evaluation of taste masking methods shows that pH dependent coacervation obtained high scoring over common methods and thus display a robust potential for industrial applications.

## Introduction

Taste masking techniques are crucial to cover the common bad taste of drugs which is critical in case of pediatric and geriatric drugs. The importance of taste masking techniques has also increased after discovering fast dissolving tablet^1–3^. The bitter taste of drugs is sensitized only when drug is dissolved in saliva and come in contact with tongue taste buds. Therefore, drugs with high solubility saliva can have more bad taste^4,5^.

Previously the taste masking was performed by adding a flavor or/and sugar, however this did not result in significant improvements as the drug molecule is unbound to flavor or sugar molecules, thus the tongue would sense the two molecules^5,6^. Therefore, Reports discussed dispersing the drug molecule in saliva-insoluble polymer to hinder saliva solubility and prevent the drug to make contacts with tongue buds. Simultaneously, the drug will be converted to amorphous phase making it highly and rapidly soluble in gastric fluids after administration. It is crucial to obtain a complete amorphization of the drug to achieve a successful taste masking^7,8^.

There are abundant reported techniques for the preparation of amorphous solid dispersions (ASD). These methods aim to obtain a drug—polymer mixture at the molecular level and include complexation, encapsulation and melt extrusion methods. Each one of these techniques has one or more disadvantages such as using costly and sophisticated instruments, complex or multistep processing, using high temperatures which causes drug degradation issues, and the use of large amounts of organic solvents causing toxicological and environmental issues^8^. One of the common complexation methods is coacervation due to its simple processing. The method involves dissolving the drug in a polymeric solution followed by gradually precipitating a drug loaded polymer either by adding anti-solvent or evaporation^1–3^. However, current coacervation methods use large amounts of organic solvents both in dissolving and precipitating steps^9^. Current work is reporting a new modified coacervation method which overcome all previously mentioned disadvantages. . . We are reporting for the first time an aqueous pH dependent coacervation method. The method includes using pH-dependent soluble drug and polymer which can be dissolved in water at selected pH level, then solid dispersion is obtained by gradually changing pH where the materials are no longer soluble.

**Figure 1:**
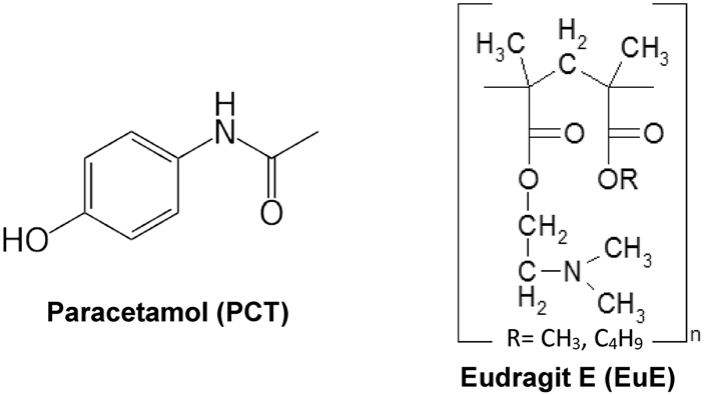
Chemical structures of PCT and EuE.

Model drug, paracetamol, and Eudragit E (Figure 1) were chosen for the preparation of solid dispersions by pH-dependent coacervation. Paracetamol (PCT) is commonly used as a model drug in taste masking methods due to its safety, low cost, and availability. Taste masking of PCT was performed via microencapsulation with polyvinyl acetate^10^, hotmelt extrusion with eudragit E^11^, forming pellets^12^, and complexation with ion-exchange resins^13^. Paracetamol (PCT) or N-acetyl-para-aminophenol, obtains a pKa value of 9.32 making it soluble in relatively acidic pH levels^7^. Eudragit E (EuE) is a cationic copolymer composed of dimethylaminoethyl, methyl, and butyl methacrylate. EuE is soluble at pH below 5.5 while it is swellable, permeable and insoluble at pH above 5.5^8^. Eudragit E is used to target drug release in acidic mediums, e.g. gastric fluid and is used for taste masking of bitter drugs^10–12^. Solubility enhancers, SLS and PEG 400 were used to improve inclusion of PCT in EuE by increasing PCT aqueous solubility during ASD preparation. Aqueous solubility of PCT can be enhanced up to two folds using 0.25–0.5% SLS^13–15^. Whereas, the solubility increases from 15 to 288 mg/mL (19 folds) in a 10:90 PEG 400 : water mixture^16,17^.

Several trials were prepared by varying PCT:EuE ratio from 1:1 to 4:1. Moreover, trials were split into two groups, SLS was added to one and PEG 400 was used for the other at concentrations of 20%, and 25% (w/w), respectively. One trial was prepared at 1:1 PCT:EuE ratio without addition of PEG 400 or SLS which was used for comparison.

Taste masking efficiency of the pH-dependent coacervation was evaluated based on its ability to produce complete ASD which ensures that PCT is completely bound to the polymer making it insoluble in saliva, thus avoiding the bitter taste. Moreover, the PCT should have acceptable release profile in gastric fluid medium. Eventually, taste of the PCT ASD should be acceptable *in vivo*. ASD of PCT and EUE is confirmed Fourier transform infra-red spectroscopy (FT-IR), Differential scanning calorimeter (DSC), and Powder X-ray diffraction (PXRD). Visual investigation of produced ASD was performed using Scanning electron microscopy (SEM). Whereas, PCT release profile was conducted using in vitro dissolution study. *In vivo* taste masking evaluation was performed tested by human volunteers^18^.

A reported scoring system, which is referred to as method technical evaluation, was applied to evaluate the pH-dependent coacervation method in comparison with previously reported taste masking methods^9^.

## Materials and methods

### Materials

Paracetamol (PCT) monoclinic FI was purchased from Sigma–Aldrich (Germany). Eudragit E 100 (EuE) polymer was obtained from Evonik Pharma Polymers (Germany). Hydrochloride acid was bought from Sigma Aldrich (Germany). Polyethylene glycol 400 (PEG 400), Sodium hydroxide and HPLC solvents were of analytical grade and purchased from Merck (Germany).

### pH-dependent coacervation method

**Figure 2:**
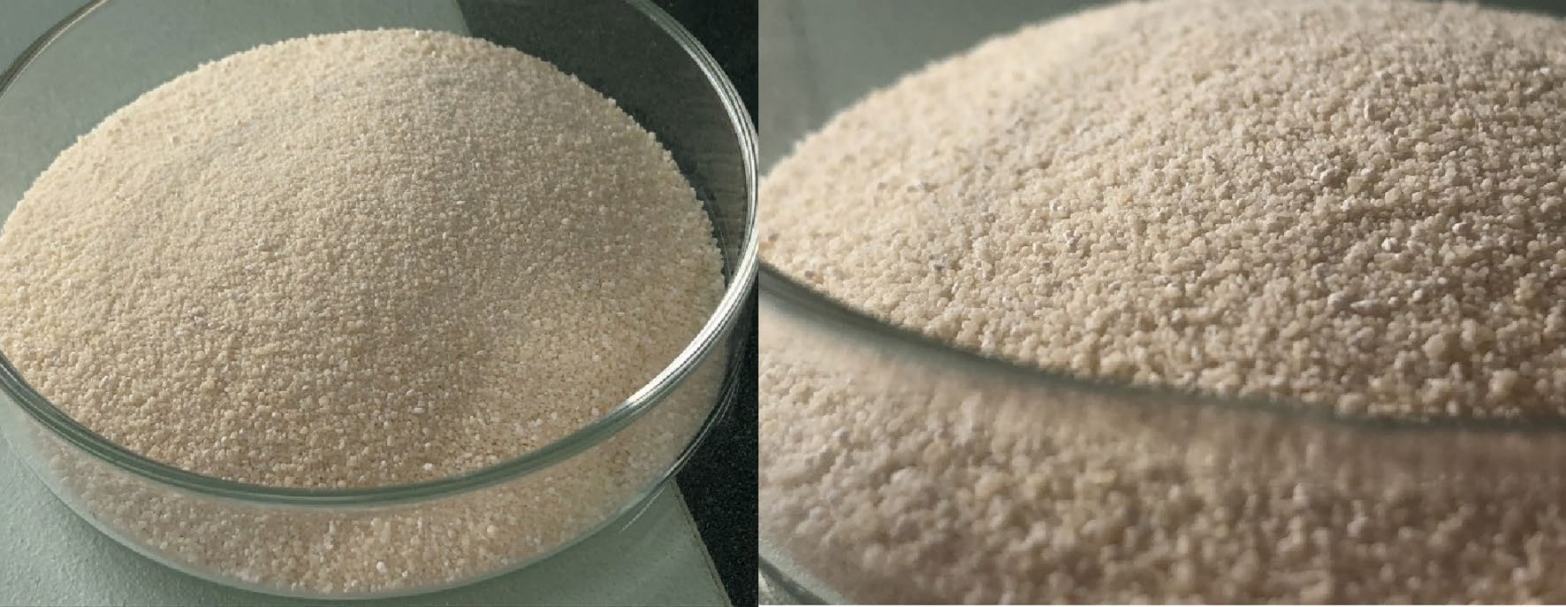
Images of produced PCT-EuE granules.

1.2 g of EuE was dissolved in a mixture of 12 mL of HCl (1 N) and 8 mL of NaOH (1 N) having a pH of 3.0. Then, SLS or PEG 400 was added to the mixture and left until obtaining clear solution. Subsequently, 3.0, 2.0 1.2 g of PCT were added and stirred until complete dissolving. Temperature was slightly increased to 40.0 °C to assist dissolving of PCT. 5 mL of NaOH (1 N) was pipetted at slowly to reach pH of 8.0 which leads to complete precipitation of EuE and PCT in form of granule. Afterwards, solid particles were filtered and washed using DI-water (ca. 10 mL) and NaOH (1 N) ca. 10 mL to remove any excess free PCT molecules. The granules were left to dry under room condition for 24 h for further analysis (Fig. 2). It is reported that 90% of PCT is present in its protonated form up to pH 7. Whereas, in alkaline pH 11, the phenol proton is removed and 90% of deprotonated form is present^19^. It is believed that during current method, reaching to pH 8 will result in deprotonation of PCT and subsequently precipitation of PCT. Moreover, stability of PCT will not be compromised at pH rage of 3–8.

8 trials were prepared with EuE and with varying SLS or PEG as additives and PCT to EuE ratio (Table 1).

**Table 1:** Composition of trials for PCT and EuE solid dispersion preparation.

#Trial	PCT (g)	EuE (g)	SLS (g)	PEG 400 (g)
T1	1.25	1.2	–	–
TS1	1.25	1.2	2.5	–
TS2	2.50	1.2	2.5	–
TS3	3.00	1.2	2.5	–
TS4	4.00	1.2	2.5	–
TP1	1.25	1.2	–	4.0
TP2	2.50	1.2	–	4.0
TP3	3.00	1.2	–	4.0
TP4	4.00	1.2	–	4.0

### Powder X-ray diffraction (PXRD) analysis

PXRD analysis was done using a Bruker D8 diffractometer with a Cu Kα radiation source tube and 1.54 Å X-ray wavelength. Emission filament voltage and amperage were 40 kV and 40 mA respectively. The scanning range of 4–40° 2θ with step size of 0.02° and reflection mode were used. Results were compared to the simulated patterns generated from the reported single crystal X-ray diffraction files from CCDC.

### Thermal analysis

Pure components, physical mixtures, and the solid dispersion trials were tested using DSC-60 plus series, Shimadzu (Japan). Samples were prepared in open aluminum pans (2–5 mg). The samples were heated at 10 C/min under nitrogen atmosphere in a temperature range between 0 and 200 °C.

### Fourier transform infrared (FT-IR) spectroscopy

FTIR spectroscopy was performed using FT/IR 4100 Jasco (Japan). FT-IR samples were prepared by compressing each sample with pure Potassium bromide (KBr) and pure KBr tablet was used as a blank for background subtraction. All spectra ranging between wave numbers 2000 and 400 cm^−1^ with scanning speed 2 mm/sec and resolution 4 cm^−1^^20–22^.

### PCT loading measurements using HPLC analysis

Paracetamol content in each trial was determined using HPLC. HPLC, Prominence, LC-2030C plus 3D, Shimadzu (Japan). Nucleodur (MN)150 mm × 3.9 mm (5 µm) at 290 nm was used for HPLC assay. The mobile phase consisted of acetonitrile/phosphate buffer (pH 3) (10:90, v/v). The flow rate was 1.5 ml/min and the retention time of paracetamol was 2.061 min. The injection volume was 20 µl.

### Scanning electron microscopy (SEM)

All samples were mounted on aluminum pin stubs using self-adhesive carbon strips (Agar Scientific, Stansted, UK). Subsequently, sample stubs were coated with 20 nm of gold using Emitech K575 sputter coater. Subsequently, SEM images acquisition was conducted using a FEI Quanta 400 scanning electron microscope (Cambridge UK) under vacuum at 5 kV and XTM microscope control software V 2.3.

### In vitro drug release studies

Drug release studies were carried out using 500 ml of 0.1 N hydrochloric acid as dissolution medium. Electrolab dissolution paddle apparatus EDT-08LX (India) at 50 rpm in temperature 37 °C was used. Samples having amounts equivalent to 500 mg of PCT were placed at each vessel. Samples then were withdrawn at five tim e intervals after 2, 5, 10, 20 and 30 min. Subsequently. samples were filtered and diluted for HPLC analysis to measure PCT concentration. Each dissolution trial was performed in triplicate^14,18^.

### Gustatory evaluation test

This test is applied on 9 volunteers where quantity of masked PCT equivalent to 100 mg of PCT is placed on the tongue of each volunteer separately for 30 s. Volunteers are asked to gargle with water immediately before and after each evaluation for 30 s. The degree of bitterness is recorded immediately as a bitterness scale ranging from 0 to 5 with being the taste of pure PCT as the highest bitter taste (its scale 5). A gap of 30 min is kept as a wash out period between the masked drug and pure drug to avoid any interference^18^. Our study protocol got an ethical approval from Damascus university research and ethics committee (REC128-9-18, date:01/04/2020).

### Technical evaluation of the preparation method

Details of the technical evaluation for taste masking product can be found in a previous report^9^. All methods have been evaluated according to the following parameters, used materials (cost and safety), equipments (cost and safety), process (cost and simplicity), and output (quality and yield). The score is calculated by adding a positive mark (+ 1) if the parameter is positive, e.g., materials used are safe or environmentally green. Otherwise, a negative mark (− 1) will be given if the parameter is negative, e.g., the materials are not safe or have an impact on environment, and (0) mark is given if no enough data is available for that parameter, e.g., the yield is not reported. The total of positives and negatives yields the final method score which is used for comparison.

## Results and discussion

### Powder X-ray diffraction (PXRD) results

**Figure 3:**
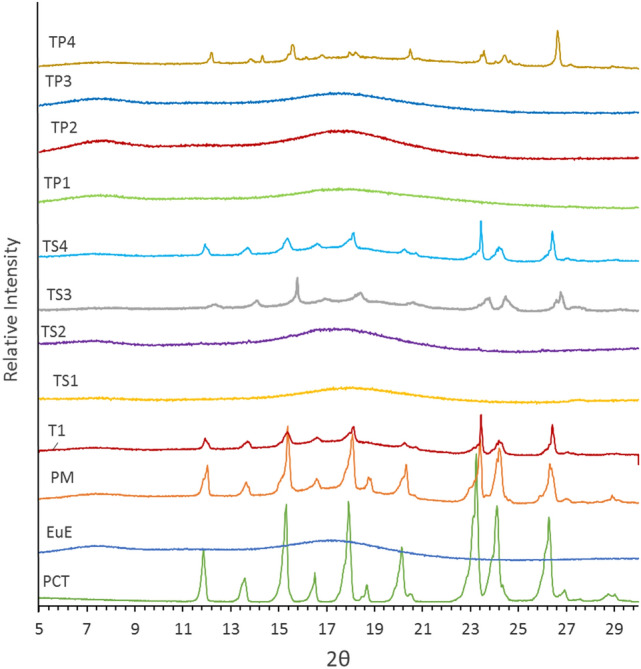
PXRD patterns of PCT, EuE, physical mixture, T1, TS, and TP trials.

According to PXRD results, trials TS1, TS2, TP1, TP2, and TP3, display no sign of crystalline PCT. While TS3, TS4, and TP4, exhibit minor content of crystalline PCT (Fig. 3).

### DSC results

**Figure 4:**
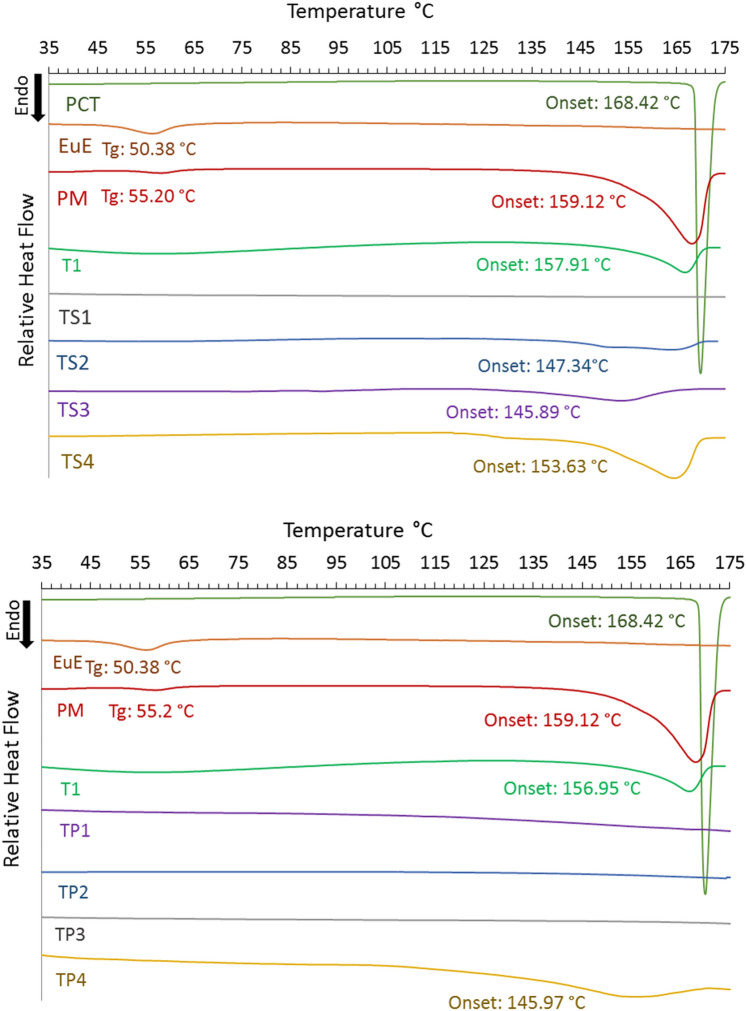
Thermograms of PCT, EUE, Physical mixture (PM), T1, and TS trials and showing peaks onsets and detected T_g_ steps.

DSC thermograms (Fig. 4) show that PCT display melting endotherm with onset of 168.42 °C, while EuE exhibit glass transition at 50.38 °C. 1:1 PM of PCT and EuE did not change Tg Significantly (55.20 °C) while PCT melting peak become broader and onset shifts to 159.12 °C. T1 still show smaller and broader endotherm shifting to 157.91 °C indicating a presence of crystalline PCT. Moreover, TS1, TS2, and TP4 show minor and more broadening melting peaks at 145.9 °C, 147.3 °C and 145.9 °C, respectively. On the other hand, TS3, TP1, TP2, and TP3 don’t display any trace of endotherms confirming no crystalline content. TS4 show significantly higher crystalline content compared to other trials with endotherm at 153.6 °C. *T*_*g*_ could not be observed at all trials thermograms.

### Fourier transform infrared (FT-IR) spectroscopy results

**Figure 5:**
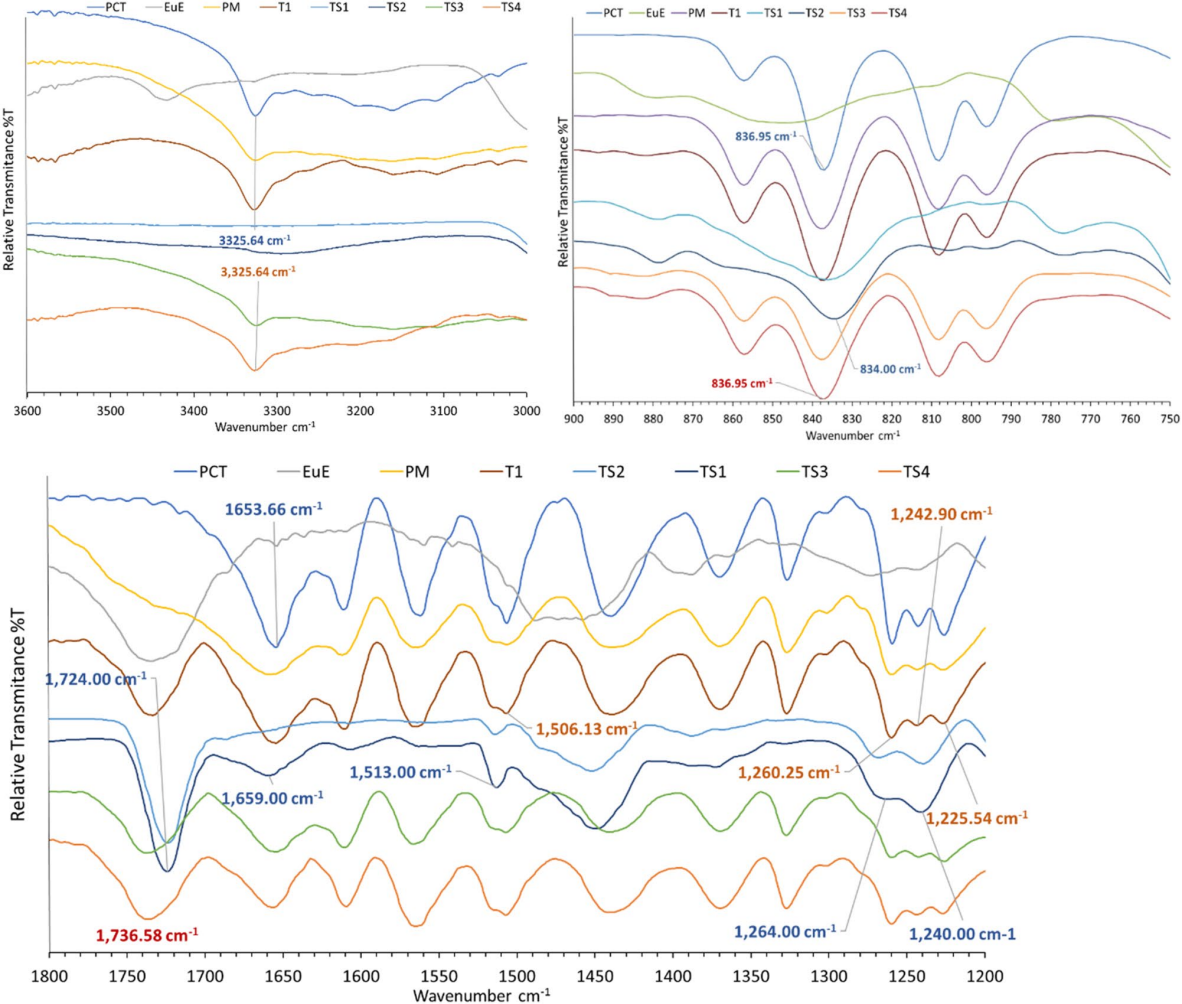
IR spectra of PCT, EuE, 1:1 PM, T1, and TS trials.

Identification of PCT-EuE ADS formation using FT-IR is reported previously^23^. PCT amorphization formation leads to a shift in 836 cm^−1^ PCT peak to 833 cm^−1^. Moreover, it was reported that PCT—EuE solid dispersion spectra are more similar to EuE except presence of additional peaks at 1514 cm^−1^. In the current work, spectra of pure PCT, EuE, 1:1 physical mixture (PM), T1, TS, and TP trials were investigated. The IR spectra of TS1, and TS2 are matching reported observations where shifting of 836.9 cm^−1^ peak of PCT to 834.0 cm^−1^ and 1513 cm^−1^ peak were observed (Fig. 5). Additional changes are shifting of PCT 1260.25 cm^−1^ peak to 1264 cm^−1^ and disappearance of a peak at 1225.54 cm^−1^. Moreover, PCT, PM, and T1 peak at 1736.5 cm^−1^ assigned to C=O stretching of EuE is shifted to 1724.0 cm^−1^ in case of TS1 and TS2. Moreover, peak disappearance and broadening of PCT –OH stretching at 3325.6 cm^−1^ and C=O stretching at 1653 cm^−1^ was observed in TS1, and TS2, respectively indicating that these groups are mostly involved in PCT-EuE hydrogen bond formation^24^. On the other hand, TS3, and TS4 spectra are identical to PM and T1 indicating a crystalline PCT content.

**Figure 6:**
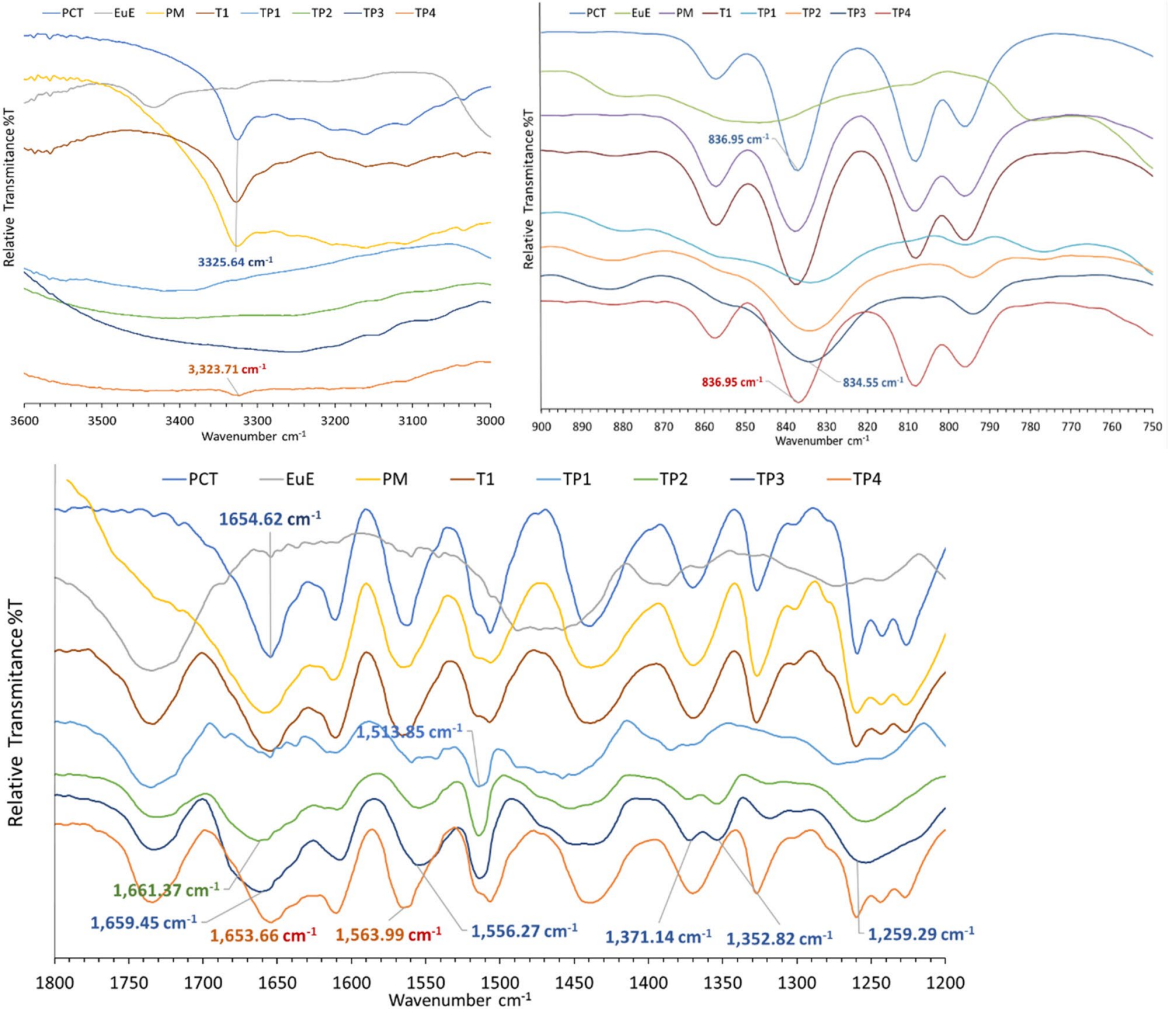
IR spectra of PCT, EuE, 1:1 PM, T1, and TP trials.

IR spectra of TP1, TP2, and TP3 trials (Fig. 6) also show shifting of PCT 836.95 cm^−1^ peak to 834.5 cm^−1^ and presence of 1512.88 cm^−1^ peak confirming formation of amorphous PCT. Additional changes were observed which did not occur in case of TS1, and TS2 trials. These changes are splitting of PCT 1368.25 cm^−1^ peak to 1371.1 and 1352.8 cm^−1^ and shifting of PCT 1563.9 cm^−1^ peak to 1556.2 cm^−1^. Peak shifting is observed in TP1, 2, and 3 compared to PM and T1 from 1563 to 1556 cm^−1^ and from 1654 to 1660 cm^−1^ assigned to PCT N–H in plane deformation and C=O stretching, respectively. Additionally, -OH stretching peak at 3325 cm^−1^ is disappeared in TP1, 2, and 3. The change in vibrational state of these groups indicate a possible hydrogen bond formation with EuE. Notably, as PCT content increases with TP 3 and 4, intensity of PCT peaks are increased. Moreover, TP4 spectrum contains peaks matching PM and T1 spectra indicating a crystalline PCT content.

FT-IR helped to confirm presence of amorphous PCT and that the absence of diffractions in PXRD or endotherm in DSC is due to amorphisation and not to absence of PCT. Moreover, FT-IR results show an agreement with both PXRD and DSC results confirming that TS1, TS2, TP1, TP2, TP3 contain amorphous PCT while T1, TS3, TS4, and TP4 contain crystalline PCT.

### PCT %loading results

HPLC results show that PCT loading% of TS1 and TS2 which contain 100% PCT amorphous is 6.57%, and 10.05%, respectively. Whereas TS3 and TS4 which obtain both amorphous and crystalline phases, their PCT loading is 30.34% and 40.65%. However, TP trails showed better PCT loading as it reached up to 17.35% in TP3 which contain 100% PCT amorphous (Table 2).

**Table 2:** PCT loading% for each trail.

Trial	PCT Loading (%)
TS1	6.57
TS2	10.05
TS3	30.34
TS4	41.65
TP1	8.36
TP2	13.51
TP3	17.35
TP4	40.34

Both SLS and PEG 400 assisted in increasing amorphous content in EuE granules by improving PCT aqueous solubility during preparation process. However, PEG achieved better loading reaching up to 17.34% of amorphous PCT while SLS assisted in achieving up to 10.05% amorphous PCT loading. This is could be explained as the ability of PEG in increasing PCT aqueous solubility is larger compared to SLS^16^.

M. Maniruzzaman, et. al have reported using Kollidon VA64 for taste masking of paracetamol using hot-melt extrusion and achieving 30% loading^20^. Moreover, M. Rajesh, et. al have achieved 25% loading using ion-exchange resin technology for taste masking of ciprofloxacin^25^. S. M. Alshehri have also achieved 20% loading of mefenamic acid—Eudragit EPO using hot-melt extrusion^26^. The optimum achieved loading for current method is 17.35%. Therefore, we can assume that pH-dependent coacervation is more suitable for lower doses unless further improvement is attempted in the future.

### Scanning electron microscopy (SEM) results

**Figure 7:**
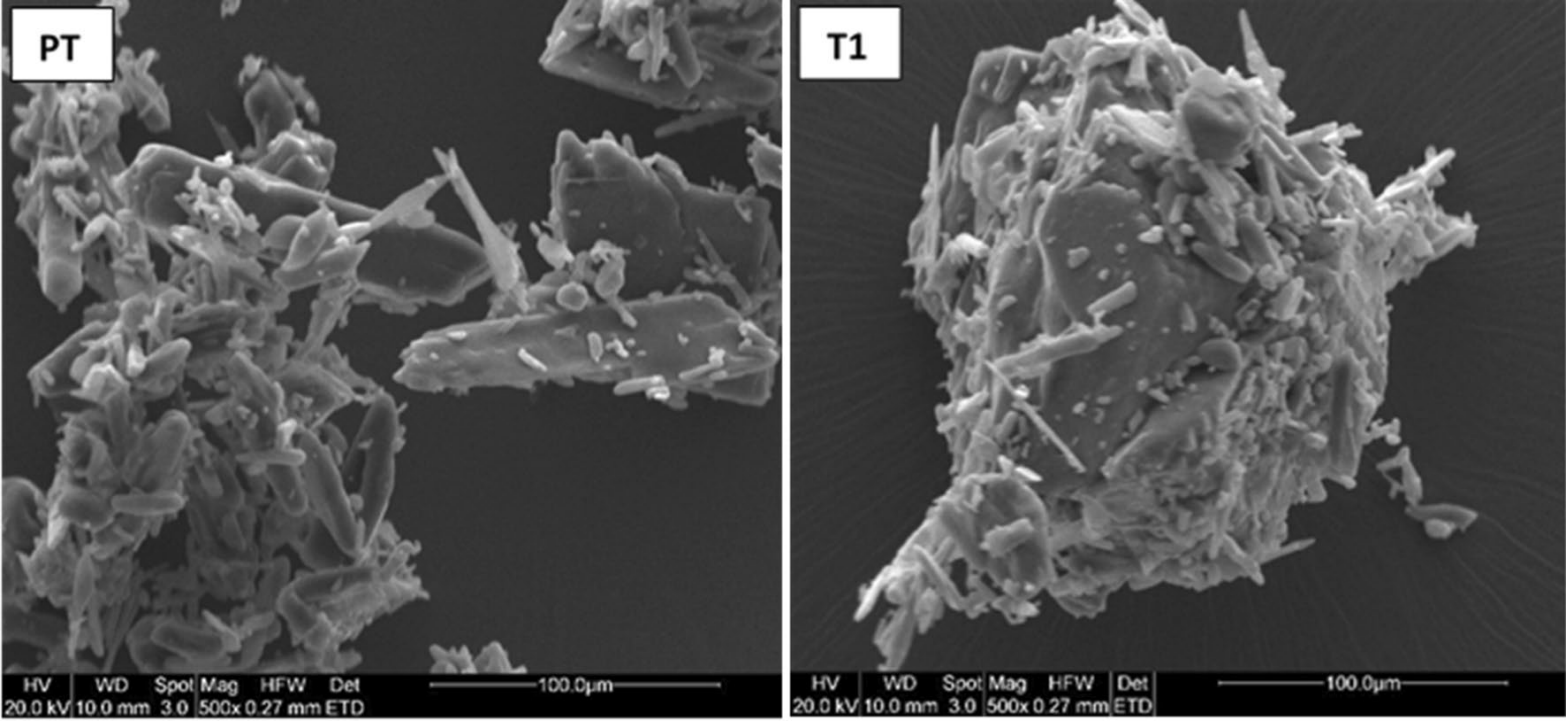
SEM images of raw PCT and T1 as a reference formula.

SEM images show that PCT crystals have needle to platelet shapes with crystal lengths ranging between 10 and 100 μm (Fig. 7). Whereas, EuE shows spherical agglomerates and the spheres have similar diameters of around 20 μm (Fig. S1, Supporting information). SEM images for T1 show formation of larger granular agglomerates of EuE having dimensions ranging from 70 to 400 μm with significant presence of PCT crystals adhered to granules surface (Fig. 7).

**Figure 8:**
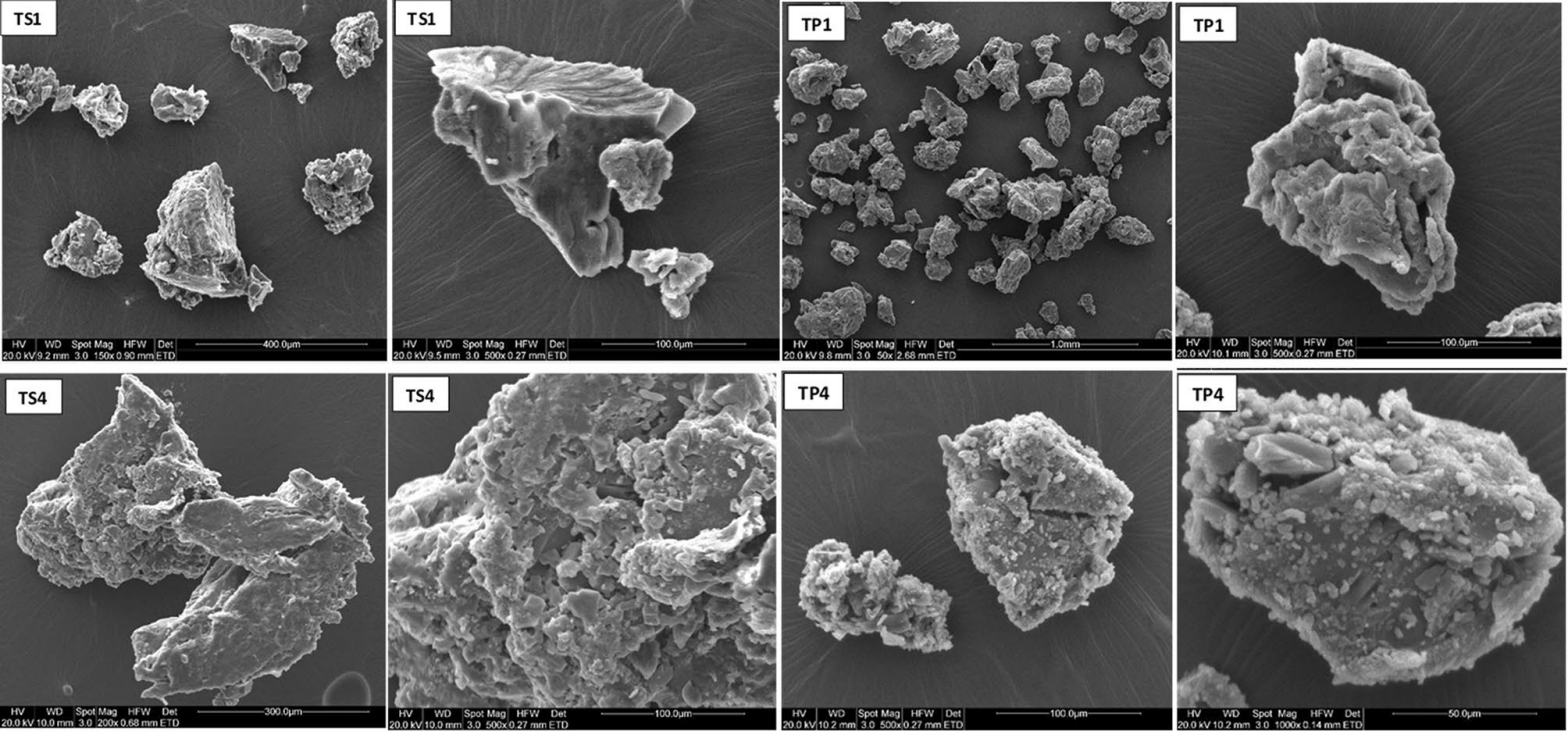
SEM images of TS1, TS4, TP1 and TP4 particles. TS1 and TP1 display smooth surface and no presence of PCT crystal is observed. However, PCT crystals can be observed on the surface of TS4 and TP4 particles.

Moreover, images of TS and TP trails show similar granules shape and size. No sign of PCT crystals was observed for TS1, TS2, TP1, TP2, and TP3. However, in case of TS3, TS4, and TP4, presence of PCT crystals can be observed (Figs. 8, S2 and S3, Supporting information).

### In vitro dissolution results:

**Figure 9:**
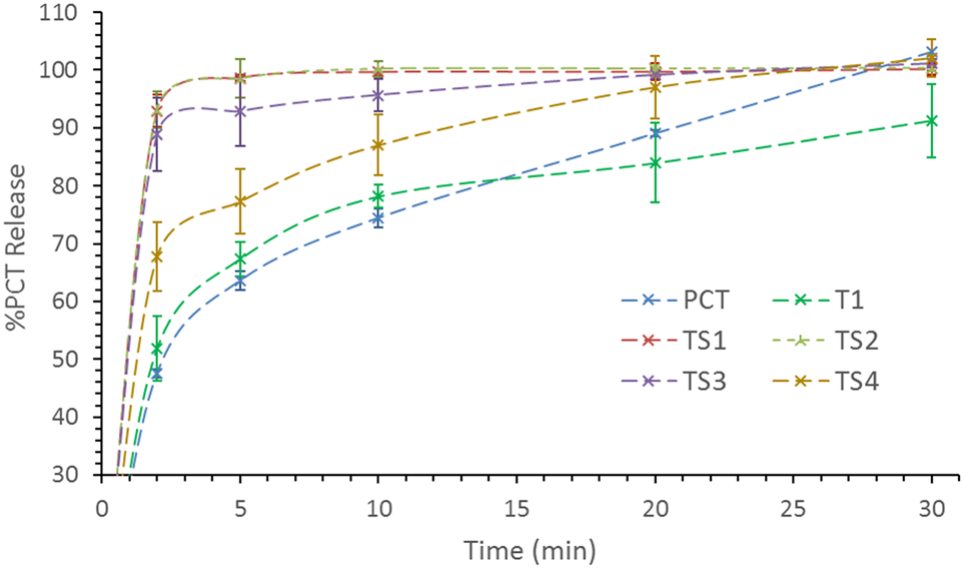
Dissolution profiles showing the %release of PCT from, pure PCT (blue), TS1 trial (red), TS2 (green), TS3 (purple), and TS4 (yellow).

Dissolution results show that pure PCT, and T1 release reached 90% after 20 min, while in TS1, TS2, it reached 90% after 2 min and in case of TS3 and TS4 after 5 and 10 min, respectively (Fig. 9, Table 3). Moreover, T1 release show that EuE alone does not assist in PCT dissolution rate especially the majority of its PCT content is crystalline. The results are in good agreement with PXRD and FT-IR which showed that TS1 and TS2 are of complete amorphous content while TS3 and TS4 showed partial PCT crystalline content which is more in TS4. This explains the delay in dissolution rate as it slows down as crystalline content increases.

**Table 3:** Dissolution %Release data of PCT in 0.1 N HCl medium.

Time (min)	PCT %Release, n = 3 ± standard deviation					
	PCT	T1	TS1	TS2	TS3	TS4
2	46.86 ± 2.89	51.81 ± 5.58	92.93 ± 2.76	93.12 ± 3.17	88.82 ± 6.30	67.70 ± 6.00
5	63.61 ± 0.71	67.31 ± 2.93	98.68 ± 0.50	98.52 ± 3.36	92.85 ± 5.89	77.29 ± 5.60
10	74.47 ± 1.64	78.17 ± 1.96	99.70 ± 0.63	100.21 ± 1.38	95.66 ± 2.78	87.05 ± 5.20
20	89.04 ± 1.61	83.97 ± 6.83	99.71 ± 1.38	100.25 ± 0.10	99.12 ± 0.59	97.03 ± 5.40
30	101.14 ± 0.54	91.27 ± 6.29	100.21 ± 1.01	100.34 ± 0.48	101.10 ± 0.74	102.10 ± 3.30

Time (min)	PCT	T1	TP1	TP2	TP3	TP4
2	46.86 ± 2.89	51.81 ± 5.58	96.28 ± 2.89	95.36 ± 4.51	90.73 ± 5.34	67.45 ± 6.36
5	63.61 ± 0.71	67.31 ± 2.93	99.26 ± 0.71	98.36 ± 2.47	94.75 ± 4.68	86.63 ± 4.85
10	74.47 ± 1.64	78.17 ± 1.96	100.02 ± 1.64	100.11 ± 1.32	97.87 ± 2.05	93.82 ± 6.79
20	89.04 ± 1.61	83.97 ± 6.83	99.95 ± 1.61	100.21 ± 0.58	99.70 ± 1.14	98.50 ± 4.13
30	101.14 ± 0.54	91.27 ± 6.29	100.20 ± 0.54	99.98 ± 1.46	101.20 ± 0.78	100.50 ± 1.50

**Figure 10:**
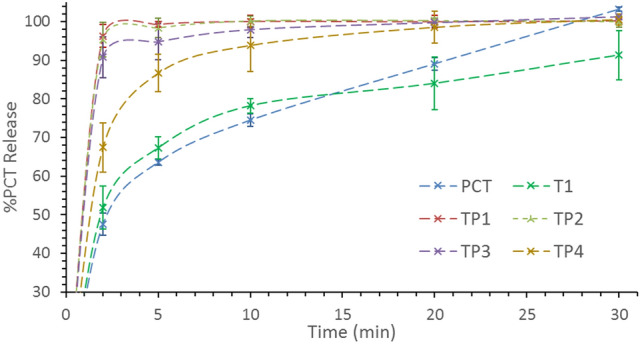
Dissolution profiles showing the %release of PCT from, pure PCT (blue), TP1 trial (red), TP2 (green), TP3 (purple), and TP4 (yellow).

The same situation applies to TP trials as in case of TP1, TP2, and TP3, the dissolution rate reached 90% after 2 min only due to complete amorphous content in these trials. On the other hand, dissolution rate of TP4 with partial crystalline content reached 90% after 5 min (Fig. 10, Table 3).

Clearly, PEG superior solubility enhancement over SLS has an impact on dissolution rate as well as PCT %loading. This is due as dissolution profiles of TP trials display faster rates compared to TS profiles. Moreover, TP4 exhibits about 90% PCT release after 5 min compared to about 90% release after 10 min in TS4.

### Gustatory evaluation test:

Trials TS1, TS2, TP1, TP2 and TP3 were given to volunteers to evaluate taste masking in comparison with pure PCT powder. All trails were almost accepted by all volunteers. Scores obtain by the volunteers show that all tested trials obtained scores of 1–2 compared to score of 5 in case of pure PCT (Table 4).

**Table 4:** Gustatory evaluation scores of trials for In vivo evaluation of PCT taste masking.

Volunteer number	Pure PCT	TS1	TS2	TP1	TP2	TP3
1	5	1	1	0	1	0
2	5	1	1	1	2	1
3	5	1	1	1	1	1
4	5	1	2	1	2	2
5	5	1	1	1	1	1
6	5	1	1	1	1	1
7	5	1	1	1	1	1
8	5	1	1	1	1	1
9	5	1	2	0	1	1

### Technical evaluation of the preparation method

Evaluation of the melting methods was discussed earlier^9^ and found that fusion and hotmelt extrusion got total scores of 1 and 3, respectively. Both methods got negative scores for equipment costs. Additionally, fusion method obtained negative scores for process cost and simplicity as it involves multiple and complex steps.

Evaluation of complexation and microencapsulation methods was also reported previously and reported final scores were found to be 5, and 3 for cyclodextrin and ion exchange complexation, respectively. Mechanical microencapsulation (Table 5) was found to obtain the best final score of 5 with higher potential for scaling up compared to cyclodextrin complexation method. Chemical microencapsulation display issues with materials and equipment safety in addition to process complexity, thus obtained a final score of 1. Moreover, physical microencapsulation obtained a final score of 3 due to process complexity and low output quality^9^.

**Table 5:** Evaluation of Taste masking methods.

Solid dispersion methods		Most used material	Evaluation									Score
			Material			Equipment		Process		Output		
			Ratio	Cost	Safety	Cost	Safety	Cost	Simple	Quality	Yield	
Melting methods	Fusion	Octadecanol	1:4	+ 1	+ 1	− 1	+ 1	− 1	− 1	+ 1	0	1
	Extrusion	Eudragit E	1:4	− 1	+ 1	− 1	+ 1	+ 1	+ 1	+ 1	0	3
Complexation methods	Cyclodextrin	Cyclodextrin	1:3	+ 1	+ 1	+ 1	+ 1	+ 1	− 1	+ 1	0	5
	Ion exchange	Tulsion-335	1:3	− 1	+ 1	+ 1	+ 1	+ 1	+ 1	− 1	0	3
Microencapsulation methods	Chemical	MADB*	1:4	+ 1	− 1	+ 1	− 1	+ 1	− 1	+ 1	0	1
	Physicochemical	Alginate	1:4	+ 1	+ 1	+ 1	+ 1	+ 1	− 1	− 1	0	3
	**pH-dep Coacervation**	**Eudragit E**	**1:2**	** + 1**	** + 1**	** + 1**	** + 1**	** + 1**	** + 1**	** + 1**	**0**	**7**
	Mechanical	Eudragit E	1:2	+ 1	+ 1	− 1	+ 1	+ 1	+ 1	+ 1	0	5

pH-dep Coacervation method is the developed method in this research so it is provided in bold.

*Methacrylic acid divinyl benzene.

For pH-dependent coacervation method, all materials are safe and cheap. No special or costly equipments are required for the preparation, which are also safe and have lower energy consumption compared to other methods. The method succeeded in producing solid dispersion with no crystalline content according to PXRD and DSC. As conclusion the final score is 7.

## Conclusion

pH-dependent coacervation method is reported here for the first time as an amorphous solid dispersion preparation method. The approach depends on the solubility dependent of the polymeric carrier, the Eudragit E. Moreover, the drugs to be used must display a proper solubility at certain pH level such as paracetamol. PXRD, DSC, and FT-IR analysis assisted in confirming complete amorphization of the drug and formation of hydrogen bonding between paracetamol and Eudragit E. The method is considered a promising method for drug taste masking as it is simple and does not require the use of organic solvents, high temperatures or sophisticated equipments. Moreover, the method overcomes most of the challenges other methods are facing including cost, safety, and environmental problems. According to a reported technical evaluation scoring system, pH-dependent coacervation can be considered the best one in preparing solid dispersion with total score of 7.

## Supplementary Information

Supplementary Information
