# Supplementary material for: Aqueous and pH dependent coacervation method for taste masking of paracetamol via amorphous solid dispersion formation
Source: Sci Rep. 2021 Apr 26;11:8907. doi: 10.1038/s41598-021-88312-6 (PMC8076289; doi:10.1038/s41598-021-88312-6)
Supplement: Supplementary file 1 — Supplementary Information [file 41598_2021_88312_MOESM1_ESM.pdf]

Supporting information - SEM images

Manuscript:

Title: Aqueous and pH Dependent Coacervation Method for Taste Masking of Paracetamol via Amorphous Solid Dispersion Formation

Authors: Basheer Al-kasmi[a], M. Bashir Alsirawan[b], Anant Paradkar[b], Abdulhakim Nattouf[a], and Hind El-zein[a].

a Department of Pharmaceutics and Pharmaceutical Technology, Faculty of Pharmacy, Damascus University, Syria

b Center for Pharmaceutical Engineering Science, University of Bradford, United Kingdom

SEM image for TS and TP formulations

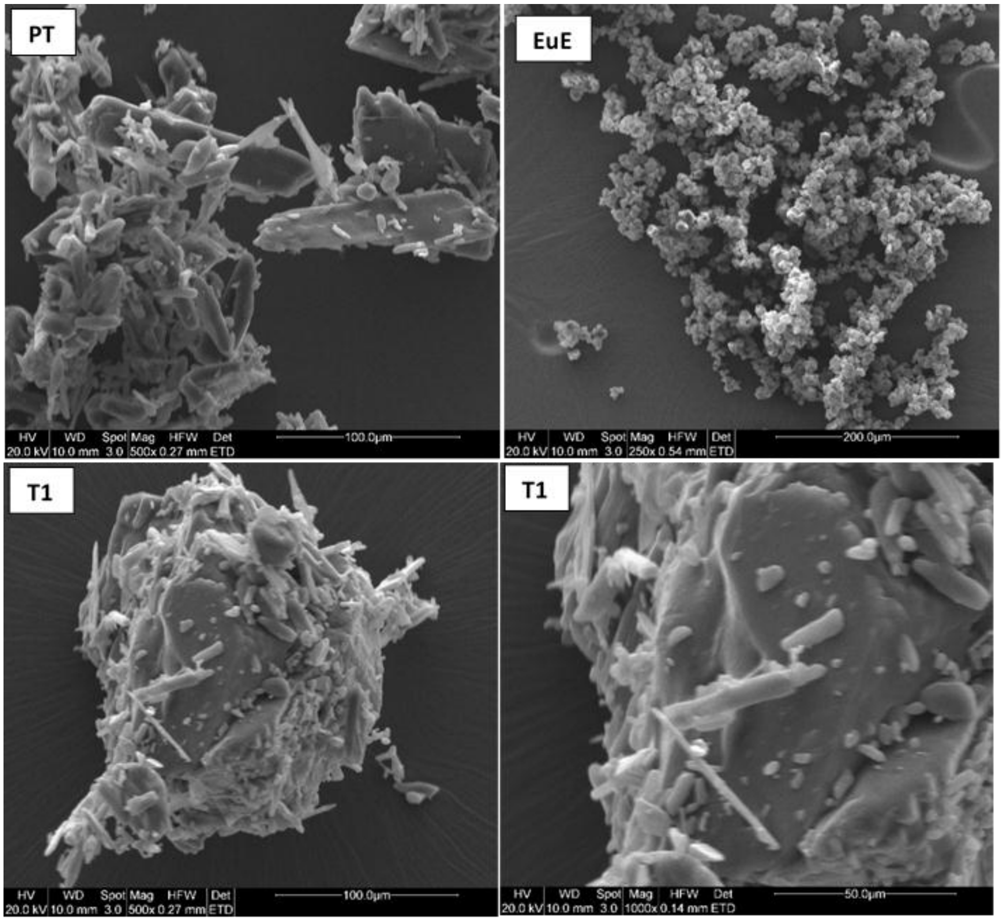

Figure S1: SEM images of raw PCT and EuD and T1.

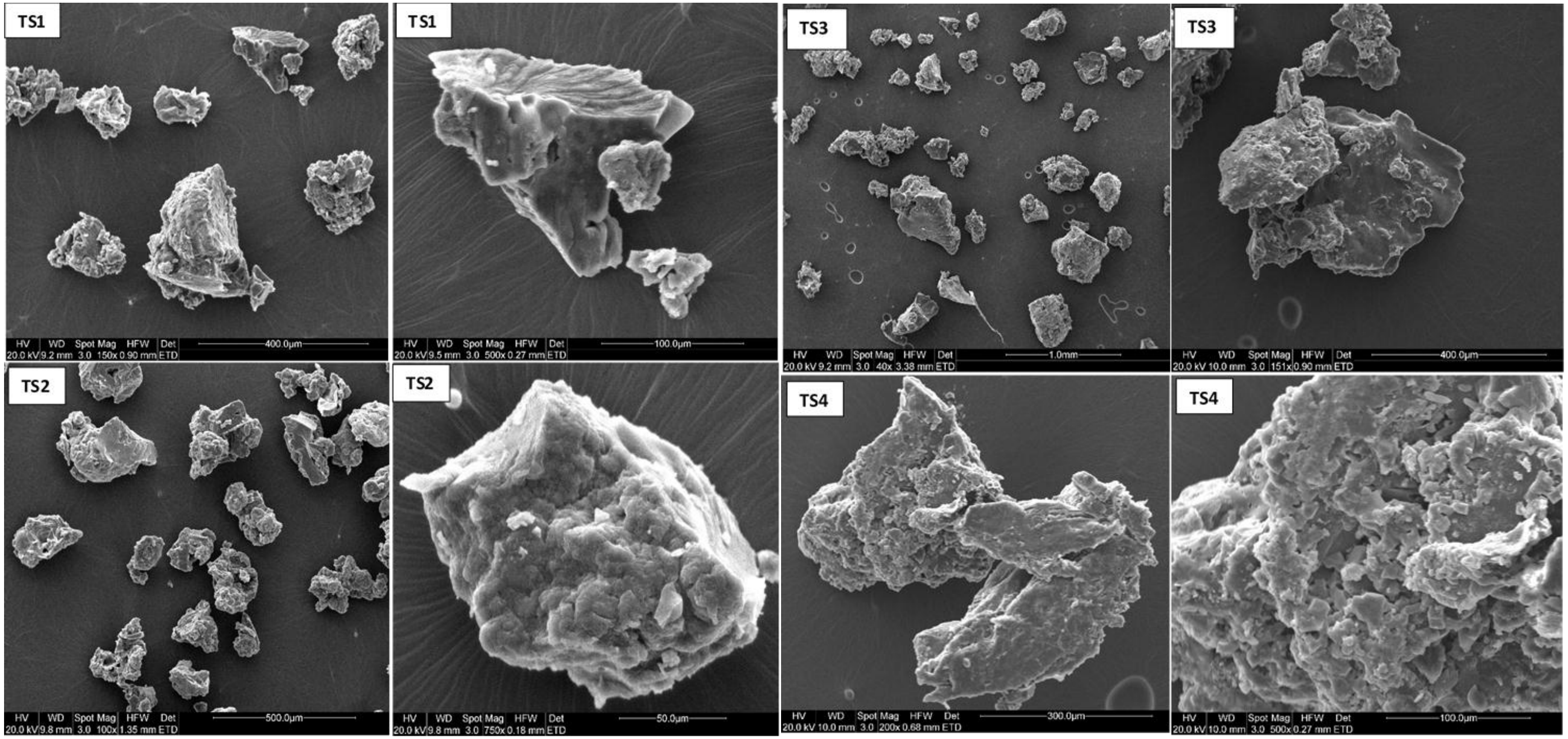

Figure S2: SEM images of TS Formulations.

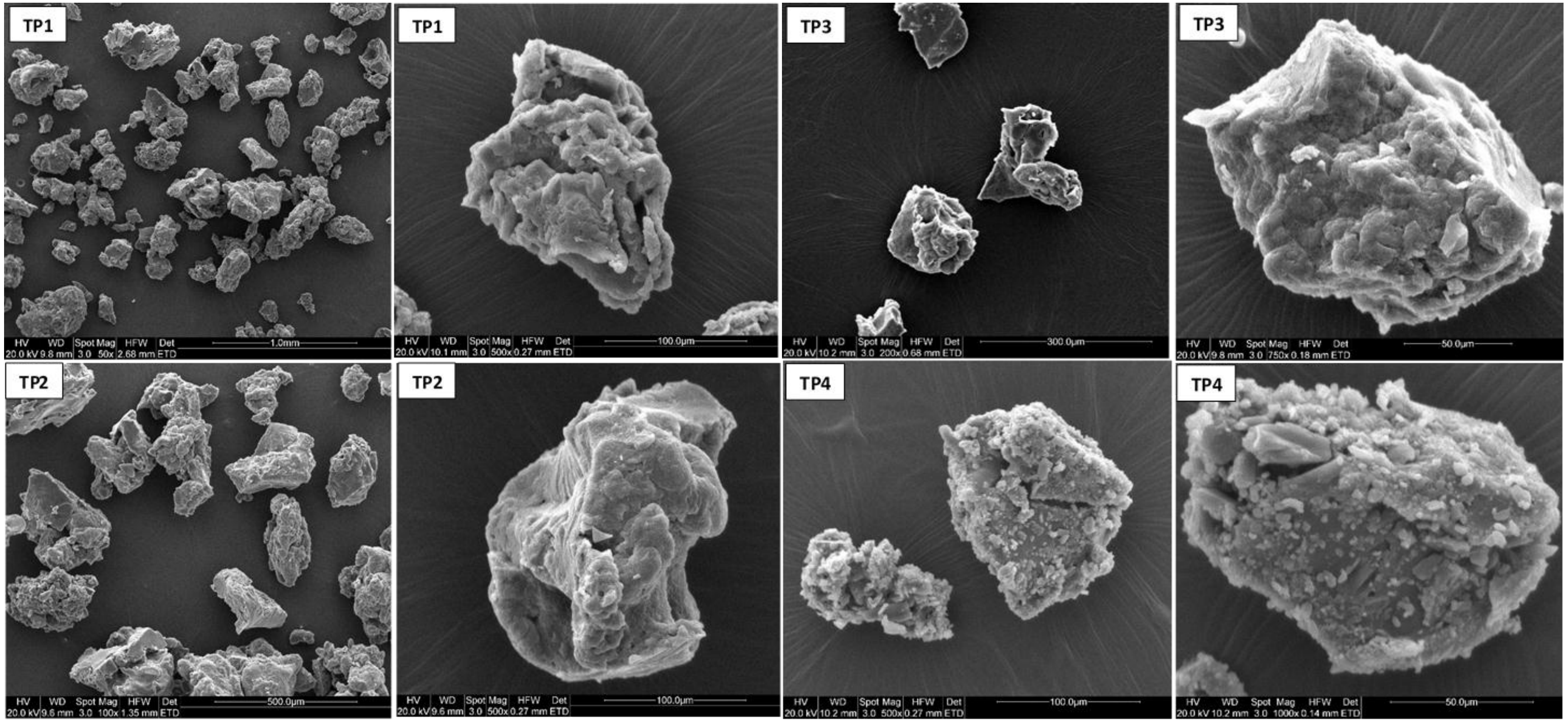

Figure S3: SEM images of TP Formulations.
